# The association of kynurenine pathway metabolites with symptom severity and clinical features of bipolar disorder: An overview

**DOI:** 10.1192/j.eurpsy.2022.2340

**Published:** 2022-11-11

**Authors:** Francesco Bartoli, Riccardo M. Cioni, Daniele Cavaleri, Tommaso Callovini, Cristina Crocamo, Błażej Misiak, Jonathan B. Savitz, Giuseppe Carrà

**Affiliations:** 1 Department of Medicine and Surgery, University of Milano-Bicocca, Monza, Italy; 2 Department of Psychiatry, Wrocław Medical University, Wrocław, Poland; 3 Laureate Institute for Brain Research, Tulsa, Oklahoma, USA; 4 Oxley College of Health Sciences, The University of Tulsa, Tulsa, Oklahoma, USA; 5 Division of Psychiatry, University College London, London, United Kingdom

**Keywords:** Bipolar disorder, clinical features, kynurenine pathway, tryptophan

## Abstract

**Background:**

The balance between neurotoxic and neuroprotective effects of kynurenine pathway (KP) components has been recently proposed as a key element in the pathophysiology of bipolar disorder (BD) and related mood episodes. This comprehensive overview explored the link of KP with symptom severity and other clinical features of BD.

**Methods:**

We searched Medline, Embase, and PsycInfo electronic databases for studies assessing the association of peripheral and/or central concentrations of KP metabolites with putative clinical features, including symptom severity and other clinical domains in BD.

**Results:**

We included the findings of 13 observational studies investigating the possible variations of KP metabolites according to symptom severity, psychotic features, suicidal behaviors, and sleep disturbances in BD. Studies testing the relationship between KP metabolites and depression severity generated mixed and inconsistent findings. No statistically significant correlations with manic symptoms were found. Moreover, heterogeneous variations of the KP across different clinical domains were shown. Few available studies found (a) higher levels of cerebrospinal fluid kynurenic acid and lower of plasma quinolinic acid in BD with psychotic features, (b) lower central and peripheral picolinic acid levels in BD with suicide attempts, and (c) no significant correlations between KP metabolites and BD-related sleep disturbances.

**Conclusions:**

An imbalance of KP metabolism toward the neurotoxic branches is likely to occur in people with BD, though evidence on variations according to specific clinical features of BD is less clear. Additional research is needed to clarify the role of KP in the etiopathogenesis of BD and related clinical features.

## Introduction

Bipolar disorder (BD) is a severe and chronic mental illness [1] with an estimated lifetime prevalence of about 2% [2]. BD is typically characterized by disabling mood fluctuations as well as, in its current conception, by an array of symptoms including sleep disturbances, psychotic features, and suicidal behaviors [1]. Pharmacological treatments of BD rely on many different agents, including mood stabilizers, antipsychotics, and antidepressants [3]. Nonetheless, the neurobiology of BD is still far from clear. The kynurenine pathway (KP), key to the metabolism of the essential amino acid L-tryptophan (TRP), is among the most studied enzymatic pathways because of its potential involvement in a range of neuroinflammatory disorders, including BD [4]. TRP is the substrate of various bioactive compounds that have many physiological roles, notably neural transmission and signaling [5, 6]. Although serotonin is its best-known metabolite, owing to its role in the pathophysiology of mood disorders [7], more than 95% of TRP is not converted into serotonin but rather metabolized along the KP [4, 6]. The KP has been investigated since the early twentieth century [8] but its importance was long thought to be linked primarily to the *de novo* synthesis of nicotinamide and, consequently, nicotinamide adenine dinucleotide, a coenzyme involved in several biological processes such as redox reactions required for mitochondrial function [4, 5, 8, 9]. Instead, no intrinsic neurobiological activity was demonstrated for the metabolites of the pathway until the late 1970s [8, 10]. Since then, interest in the KP has grown gradually [8], and research has led to the discovery that many of the metabolites generated along the pathway—collectively known also as “kynurenines (KYNs)” or “TRP catabolites”—are physiologically active and involved in inflammation, immunoregulation, and brain function [4, 6, 8]. Thus, the KP has attracted the attention of disparate disciplines [8], including psychiatry, because of its potential role in the etiopathogenesis of a number of diseases [4, 6].

**Figure 1.**
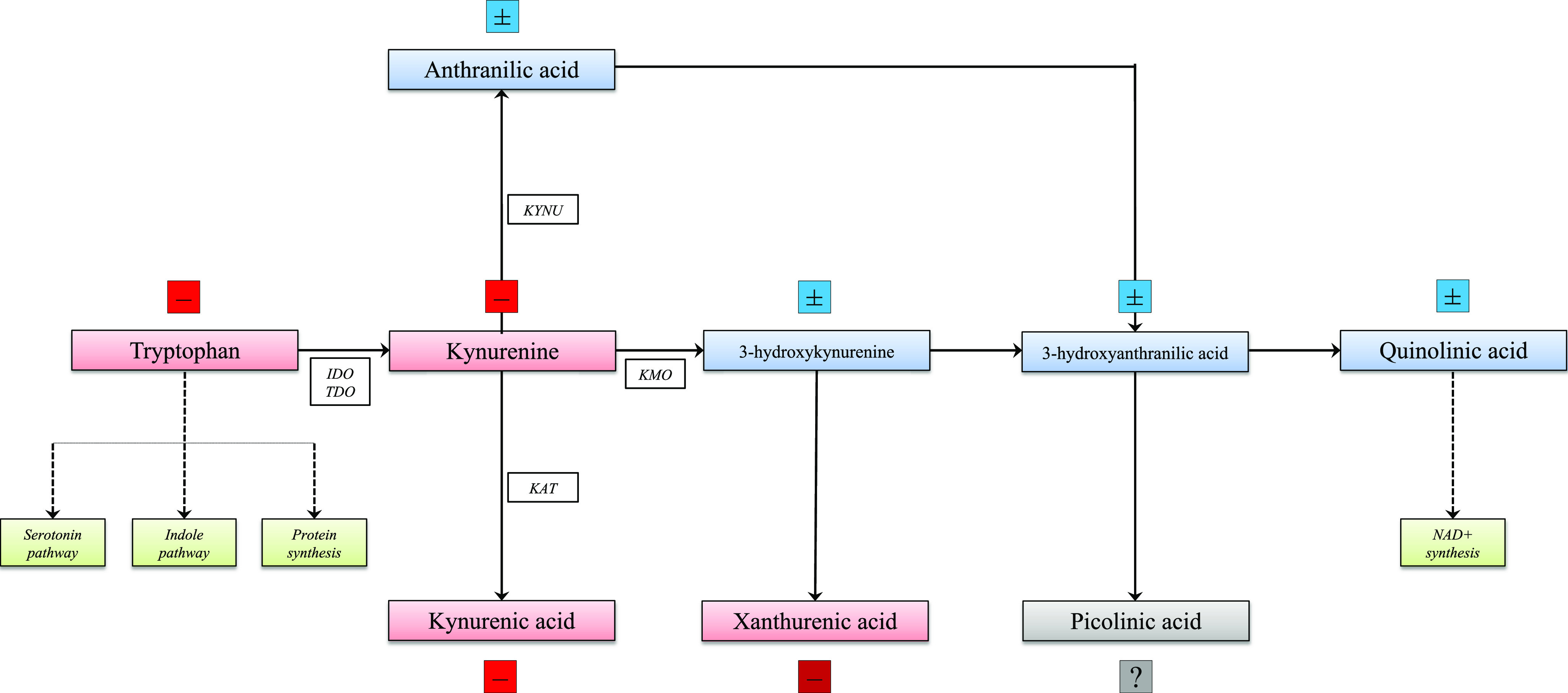
Schematic representation of the kynurenine pathway and related blood variations in bipolar disorder. –, decrease in bipolar disorder (red); ±, no variations in bipolar disorder (blue); ?, unclear variations in bipolar disorder (gray). Abbreviations: IDO, indoleamine 2,3-dioxygenase; KAT, kynurenine aminotransferase; KMO, kynurenine 3-monooxygenase; KYNU, kynureninase; NAD+, nicotinamide adenine dinucleotide; TDO, tryptophan 2,3-dioxygenase.

An overview of the KP is reported in Figure 1. In brief, the enzyme indoleamine 2,3-dioxygenase (IDO), with its two isoforms (IDO1 and IDO2), transforms TRP into KYN in the immune system and the brain, while tryptophan dioxygenase is responsible for the same reaction in the liver. The KYN/TRP ratio in the blood thus describes the activity of IDO and can be used as a proxy for the conversion of TRP into KYN. KYN is in turn catabolized into different molecules including kynurenic acid (KYNA), anthranilic acid (AA), 3-hydroxykynurenine (3HK), xanthurenic acid (XA), 3-hydroxyanthranilic acid (3HAA), quinolinic acid (QA), and picolinic acid (PA) [11]. The main branch of the cascade leads to 3HK, 3HAA, and QA (the so-called “QA branch”), whereas KYNA and XA are formed in competing branches of the pathway [4]. Kynurenine 3-mono-oxygenase catabolizes KYN into 3HK leading it down the QA branch, hence its inhibition leads to the accumulation of KYN and increases its catabolism toward the production of KYNA via the KYN aminotransferase (KAT) isozymes (KAT-2 in the brain) whose activity is mirrored by the KYNA/KYN ratio [12, 13].

KP metabolites are putatively neuroactive, theoretically modulating neuroplasticity and influencing NMDA receptor signaling and glutamatergic neurotransmission [4]. For example, KYNA seems to have a neuroprotective role by antagonizing the excitotoxic effect of QA and competitively inhibiting ionotropic glutamate receptors in order to attenuate activity at the glycine co-agonist site of the NMDA receptor [4, 8]. Other potentially neuroprotective components of the pathway include XA [14], AA [8], and PA [15]. Conversely, QA has been proven to be neurotoxic through a variety of mechanisms that include NMDA agonism with associated oxidative stress, lipid peroxidation, and interference with glutamatergic transmission [16].

The balance between neurotoxic and neuroprotective effects of KP metabolites has led to several different, and not always consistent, hypotheses concerning their role in the pathophysiology of BD. In particular, a few systematic reviews and meta-analyses have been recently published, highlighting significant variations of the KP in BD and related mood episodes, involving blood TRP, KYN, KYNA, and XA [17–20] (Figure 1). Notwithstanding this body of evidence, whether specific clinical features of BD might be linked to the peripheral and central levels of KP metabolites remains unknown. This work is thus aimed at providing a comprehensive overview synthesizing available evidence on the association between variations of the KP and BD clinical features.

## Methods

We performed an overview of research exploring the possible link between the KP and BD-related symptom severity and other clinical features, following standard methods set to report nonquantitative and narrative syntheses [21, 22]. Medline, Embase, and PsycInfo electronic databases (via Ovid) were systematically searched for articles published up to August 2022. The following search phrase was used: “*(tryptophan OR kynurenine OR kynurenic OR anthranilic OR quinolinic OR picolinic OR xanthurenic) AND (bipolar OR mania OR manic)*” as multiple purpose search in title, abstract, heading words, and keywords. We also explored the reference list of our recent systematic review and meta-analysis in this field [18]. No language or publication date restrictions were applied. We included studies that explored the association of peripheral and/or central concentrations of KP metabolites (TRP, KYN, KYNA, AA, 3HK, XA, 3HAA, QA, and PA), or their ratios, with clinical features of BD. To improve the consistency and comparability of data, we excluded studies that provide mixed data for subjects with BD and individuals with other psychiatric diagnoses. Moreover, we excluded “gray” literature, conference abstracts, dissertations, and all publications not having undergone a peer-review process. After a preliminary screening based on titles and abstracts, full texts were retrieved to evaluate eligibility. Articles were independently screened and read in full text by three authors (R.M.C., D.C., and T.C.). Any disagreement was resolved by discussion with the other authors.

## Results

Our search generated 1,808 articles (483 from Medline, 963 from Embase, and 362 from PsycInfo) and, after removing duplicates, 1,144 studies were screened. Despite the wide variability in terms of study design, single hypotheses tested, and characteristics of included samples, we included in this overview 13 observational studies [23–35]. Clinical features of BD assessed in this body of evidence were symptoms (depression and mania) severity [23–28, 30], suicidal behaviors [29, 31, 34], psychotic features [24, 31–33], and sleep disturbances [26, 35]. The characteristics of the studies included in this overview are reported in Table 1.

**Table 1.** Characteristics of included studies.

Study	Country	Bipolar disorder			Biological substrate	Tested metabolites	Tested clinical domains of bipolar disorder	Main results
		Sample size	Mean age	% males				
Benevenuto et al. [28]	USA	49	13.5	49.0	Blood	TRP; KYN; KYNA	Depressive symptom severity	Negative correlation with KYN and KYN/TRP ratio; positive correlation with KYNA/KYN ratio
							Manic symptom severity	No statistically significant correlation with any KP metabolite
Brundin et al. [34]	Sweden	21	37.5	–	Blood	PA; QA	Suicidal behavior	Lower levels of PA among suicide attempters versus healthy controls
Comai et al. [27]	Italy	66	47.6	33.3	Blood	TRP; KYN	Depressive symptom severity	Negative correlation with TRP
Fellendorf et al. [35]	Austria	226	43.9	48.2	Blood	TRP; KYN	Sleep disturbances	Negative correlation with KYN and KYN/TRP ratio ^a^
Maget et al. [25]	Austria	156	44.1	54.5	Blood	TRP; KYN; KYNA; 3HK; 3HAA	Depressive symptom severity	Negative correlation with KYNA/KYN ratio
							Manic symptom severity	No statistically significant correlation with any KP metabolite
Mukherjee et al. [26]	USA	21	36.1	52.4	Blood	TRP; KYN	Depressive symptom severity	Positive correlation with KYN and TRP
							Manic symptom severity	No statistically significant correlation with any KP metabolite
							Sleep disturbances	No statistically significant correlation of total sleep time with any KP metabolite
Myint et al. [30]	Korea	39	37.6	38.5	Blood	TRP; KYN; KYNA; 3HAA	Manic symptom severity	No statistically significant correlation with any KP metabolite
Olsson et al. [32]	Sweden	55	39.0	38.2	CSF	KYNA	Psychotic features	Higher levels of KYNA among subjects with a history of psychosis
Savitz et al. [23]	USA	63	38.8	19.0	Blood	KYN; KYNA; 3HK; QA	Depressive symptom severity	Positive correlation with 3HK
Sellgren et al. [33]	Sweden	76	–	–	CSF	KYNA	Psychotic features	Higher levels of KYNA among subjects with a history of psychosis
Sellgren et al. [31]	Sweden	163	34 ^b^	39.3	Blood	KYNA	Psychotic features	No statistically significant differences in KYNA levels
							Suicidal behaviors	No statistically significant association between KYNA and suicide attempt or self-harm
		94	36 ^b^	41.5	CSF		Psychotic features	Higher levels of KYNA among subjects with a history of psychosis
							Suicidal behaviors	No statistically significant association between KYNA and suicide attempt or self-harm
Trepci et al. [29]	Sweden	101	43.0	39.6	CSF	TRP; KYN; KYNA; PA; QA	Depressive symptom severity	No statistically significant correlation with any KP metabolite
							Manic symptom severity	No statistically significant correlation with any KP metabolite
							Suicidal behavior	Higher TRP levels and lower PA and KYN/TRP ratio in subjects with a history of suicidal behavior
Van den Ameele et al. [24]	Belgium	67	43.1	41.8	Blood	TRP; KYN; KYNA; 3HK; QA	Depressive symptom severity	Negative correlation with KYNA
							Manic symptom severity	No statistically significant correlation with any KP metabolite
							Psychotic features	Lower QA levels among subjects with a history of psychosis

*Abbreviations:* 3HAA, 3-hydroxyanthranilic acid; 3HK, 3-hydroxikynurenine; CSF, cerebrospinal fluid; KYN, kynurenine; KYNA, kynurenic acid; PA, picolinic acid; QA, quinolinic acid; TRP, tryptophan.

a Tested in 204 subjects.

b Median.

### Kynurenine pathway and depressive symptoms severity

Observational studies testing the relationship between peripheral KP metabolites and depression severity generated mixed findings [23–28]. In the majority of studies, no significant correlations between most KP metabolites and depressive symptoms were found. Savitz et al. [23] found that 3HK (but not other KP metabolites) was correlated with depression severity among 63 individuals with BD. In addition, van den Ameele et al. [24] reported a negative—albeit weak—correlation between peripheral KYNA concentrations and depression severity in a sample of 67 individuals with BD. No statistically significant correlations with depression severity were found for TRP, KYN, 3HK, and QA. Maget et al. [25] showed a negative correlation between Hamilton Depression Rating Scale (HDRS) scores and KYNA/KYN ratio (as a proxy of KAT activity) among 156 subjects with BD. Moreover, Mukherjee et al. [26] found that depressive symptom severity was significantly associated with both KYN and TRP in a sample of 21 individuals with BD, when total sleep time and BMI were accounted for. Conversely, among 66 participants with bipolar depression, Comai et al. [27] showed a negative correlation of HDRS scores with TRP. In addition, data on 49 children and adolescents with BD [28] showed that depressive symptoms were negatively correlated with KYN and the KYN/TRP ratio, and positively correlated with the KYNA/KYN ratio. Finally, the only study testing CSF in 101 individuals with BD did not find any statistically significant association of different KP metabolites with depressive symptoms [29].

### Kynurenine pathway and manic symptom severity

Data on the relationship between peripheral KP metabolites and manic symptom severity were available from five studies [24–26, 28, 30], including one on children and adolescents [28] and four on adults with BD [24–26, 30]. None of these studies could show any statistically significant correlation between manic symptoms, as measured by the Young Mania Rating Scale [24, 25, 28, 30] or the Clinician-Administered Rating Scale for Mania [26], and different KP metabolites. Similarly, the only study testing central levels of KP metabolites did not show any relevant correlation with manic symptoms [29].

### Kynurenine pathway and psychotic features

Four studies [24, 31–33] explored the relationship of psychotic features with peripheral and/or central KP metabolites in BD. In the study by Sellgren et al. [31], KYNA was found to be increased in CSF—but not in plasma—in individuals with BD and a history of psychotic features. Results confirmed findings from BD subjects belonging to the same cohort [32, 33], showing a significant association between a history of psychosis and CSF levels of KYNA during euthymia. Finally, van den Ameele et al. [24], assessing 67 subjects with BD, found decreased plasma QA in a subgroup of participants with lifetime psychotic features, although no KP metabolites were significantly correlated with Positive and Negative Syndrome Scale.

### Kynurenine pathway and suicidal behaviors

The possible link between the KP and suicidality in BD has been addressed in three studies so far [29, 31, 34]. Brundin et al. [34] reported that plasma levels of PA in 21 subjects with BD who attempted suicide were lower than in 29 healthy individuals, whereas no differences in QA concentrations were found. In a later study, Sellgren et al. [31] explored the peripheral and central concentrations of KYNA in relationship to lifetime suicide attempt or self-harm in individuals with BD: neither blood nor CSF KYNA levels differed from those of BD subjects without such a history. Nonetheless, higher CSF KYNA in subjects with suicide attempts, when compared with healthy controls, was found. Finally, in a study from the same research group [29], TRP levels were found to be higher in participants with a history of suicidal behavior compared to subjects without a similar history, whereas PA levels and the KYN/TRP ratio were found to be lower. No significant differences were found as for the other biomarkers addressed (KYN, KYNA, QA, and the PA/QA ratio) between the two groups.

### Kynurenine pathway and sleep disturbances

Two studies provided data on the relationship between KP metabolites and sleep [26, 35]. One study tested the relationship between KP and sleep [26], measuring TRP, KYN, and the KYN/TRP ratio in 21 subjects with BD. No associations between TRP, KYN, or the KYN/TRP ratio and total sleep time were found in any of the two groups. Consistently, Fellendorf et al. [35] did not find any correlation between TRP and the insomnia HDRS items in subjects with BD, even though a negative association of both KYN and the KYN/TRP ratio with difficulties falling asleep was found.

## Discussion

### Summary and interpretation of findings

In recent years, several studies have explored the potential role of KP metabolites as possible measurable biomarkers of BD, analyzing their links with relevant clinical features of the disorder. Nonetheless, evidence in this field generated mixed findings, not allowing us to draw firm and consistent conclusions. In particular, none of the studies included in our overview could find any statistically significant correlations between KP metabolites and manic symptoms, and studies correlating depression symptom severity and the KP showed heterogeneous findings involving different KP metabolites in adults and youths with BD. In particular, results pointed toward correlations in different directions between depressive symptom severity and blood TRP, KYN, and KYN/KYNA. Inconsistent evidence, based on a limited number of studies, was also reported about the relationship between alterations in KP metabolism and different clinical domains such as psychotic features, suicidal behavior, and sleep disturbances. First, evidence regarding the association between the KP and psychotic features in BD shows a selective increase of KYNA in CSF—but not in plasma—and a possible decrease in plasma QA. However, these findings are at best to be replicated, considering that they were derived from similar samples by the same research group [31–33], and only one study tested the relationship between other KP metabolites and psychotic features [24]. Second, few and small studies testing the possible relationship between the KP and suicidality in BD highlighted that a history of suicidal behavior might be associated with an imbalance of KP metabolites. In particular, higher TRP and KYNA levels, lower PA concentrations, and the KYN/TRP ratio in CSF [29, 31], as well as lower blood levels of PA, compared with healthy controls [34] were found. Finally, few data are available on sleep disturbances, a common occurrence in BD. Sleep may alleviate neuroinflammation, promoting the cellular clearance of brain metabolic toxins [36]. Consistently, sleep deprivation might activate the enzymatic degradation of TRP and a subsequent increase of neurotoxic metabolites including KYNA [37]. However, available studies do not show consistent correlations between total sleep time and KP metabolites, even though KYN and the KYN/TRP ratio might be associated with some sleep-related subdomains [35].

An important point to address, considering the paucity of data on drug-free or drug-naïve individuals, is the possible confounding role of pharmacological treatment on the relationship between the KP and different clinical features of BD. Indeed, studies addressing changes in KP metabolites suggest that psychoactive drugs may influence KP metabolism. For example, lithium, a highly pleiotropic agent, interacting with several different molecular targets, may counteract TRP catabolism by inhibiting the inflammation-induced TRP breakdown [38]. Consistently, a recent study estimated an association of poorer response to lithium with higher levels of KYN, the KYN/TRP ratio, and QA, which could indicate a pro-inflammatory state with a higher degradation of TRP toward the neurotoxic branch [39]. In addition, other mood stabilizers, such as valproate [24] and lamotrigine [29], might influence peripheral and central levels of KP metabolites in BD. Another key issue making even more complex the relationship between KP metabolites and clinical features seems the reciprocal influence of inflammation and the KP. Indeed, a heterogeneous pathogenesis of BD has been suggested, with inflammatory abnormalities and a potential response to drugs with anti-inflammatory properties [40], possibly occurring in specific subsets of patients [41, 42]. Preclinical evidence has shown that inflammation can significantly shunt TRP metabolism toward the KP through the upregulation of the expression and activity of key enzymes of the cascade [8]. In clinical studies on BD, significant correlations between the KYN/TRP ratio—a proxy measure of IDO activity—and TNF [24], C-reactive protein [43], and body mass index [44] have been shown. For instance, body mass index is an important factor influencing KP metabolites [45], and obese people with BD might represent a distinct immune-metabolic population [44]. Thus, the potential role of immune-metabolic abnormalities should be considered in the interpretation of findings on BD clinical features and the KP. In addition, also structural brain changes involving white matter (WM) in people with BD [46] might be correlated with the KP. Neuroimaging research has shown that higher levels of KYNA, which putatively protects from glutamate excitotoxicity, could exert a neuroprotective effect on WM microstructure [47]. Similarly, the neuroprotective KYNA/3HK ratio seems associated with hippocampal and amygdalar volumes in BD [23], and the KYN/TRP ratio negatively with corpus callosum microstructure integrity, amygdala volume, and cortical thickness in the frontoparietal regions [48]. Additional studies should thus address if specific neurostructural and neurofunctional alterations might correlate with KP metabolites. Finally, an additional consideration is needed about the complex relationship between central and peripheral levels of KP metabolites in BD [49]: the poor concordance between them outlines the need for additional research to determine the validity of blood assessment as a proxy marker for CNS processes. This could at least partially explain the inconsistency generated by evidence in this field so far.

### Limitations

The interpretation of findings synthesized in this overview requires caution considering some important limitations. First, based on the available literature, our review included a heterogeneous body of evidence not allowing us to perform any quantitative synthesis of the available data. Second, despite running a rigorous search aiming at providing a thorough overview of the topic, the narrative nature of our synthesis precluded stronger evidence-based inferences [50]. Third, several metabolites of the KP—namely XA, AA, 3HAA, and PA—have been poorly studied in people with BD so far, limiting the comprehensiveness of our overview. Finally, the eligible studies did not assess differences between participants in terms of other important clinical characteristics of BD, including different stages of the disease, specific features such as anxiety and mixed states [51], and psychiatric and substance-related comorbid conditions, which are highly prevalent in BD [52–54] and might be correlated with KP abnormalities [55].

## Conclusions

Although an imbalance of KP metabolism toward the neurotoxic branches in BD has been previously suggested, the evidence on variations of KP metabolites according to depressive and manic symptom severity as well as other clinical features is limited so far. Additional research, focusing on both blood and CSF concentrations of KP metabolites and taking into account also the BD-related immune-inflammatory and brain integrity burden, is needed. This would be helpful to address if variations of the KP, standing at the crossroads of monoaminergic, glutamatergic, and immune mechanisms of affective disorders, may represent a novel approach to understand etiopathogenesis and illness burden of BD.

## Data Availability

The data that support the findings of this study are available from the corresponding author upon reasonable request.
